# Extramedullary plasmacytoma (EMP): Report of a case manifested as a mediastinal mass and multiple pulmonary nodules and review of literature

**DOI:** 10.1186/1477-7819-5-123

**Published:** 2007-10-27

**Authors:** Shi-Ping Luh, Yih-Shyong Lai, Chung-Hong Tsai, Thomas Chang-Yao Tsao

**Affiliations:** 1 Department of Surgery, Chung-Shan Medical University and Chia-Yi Christian Hospital, Taichung City, Taiwan; 2 Pathology, Chung Shan Medical University and Hospital, Taichung City, Taiwan; 3 Thoracic Medicine, Chung Shan Medical University and Hospital, Taichung City, Taiwan

## Abstract

**Background:**

Extramedullary plasmacytoma (EMP) is a rare plasma cell neoplasm of soft tissue without bone marrow involvement or other systemic characteristics of multiple myeloma

**Case presentation:**

A 42 year-old woman presented with intermittent dry cough of 10 months duration. Her breathing sound was slightly coarse without rales or rhonchi on auscultation. CT scan revealed a right anterior mediastinal shadow with multiple pulmonary nodular lesions. A video-assisted thoracoscopic surgery (VATS) was performed. Histopathology showed it to be a myeloma.

**Conclusion:**

This is the first presentation of EMP with a mediastinal mass with multiple pulmonary nodules.

## Background

Extramedullary plasmacytoma (EMP) is a plasma cell neoplasm of soft tissue without bone marrow involvement or other systemic characteristics of multiple myeloma [1-3]. It is rare and mainly involves the upper aero-digestive tract. Herein we report an extremely unusual presentation as a mediastinal mass and multiple pulmonary nodules, but without bone marrow involvement or other characteristics of multiple myeloma.

## Case presentation

**Figure 1:**
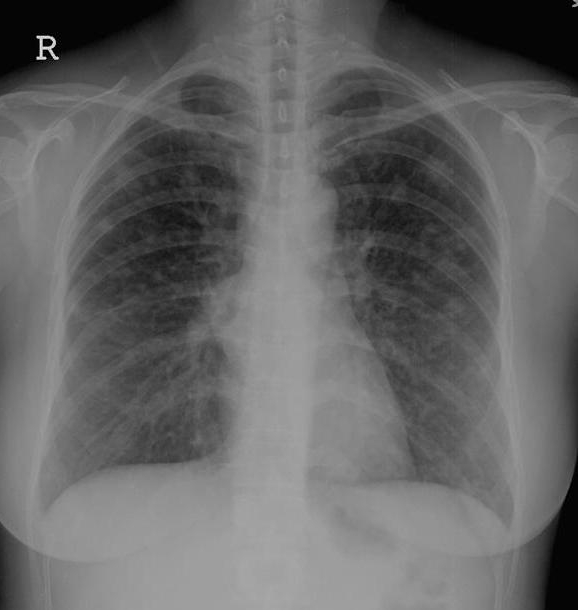
The chest X-ray revealed multiple pulmonary nodular lesions.

**Figure 2:**
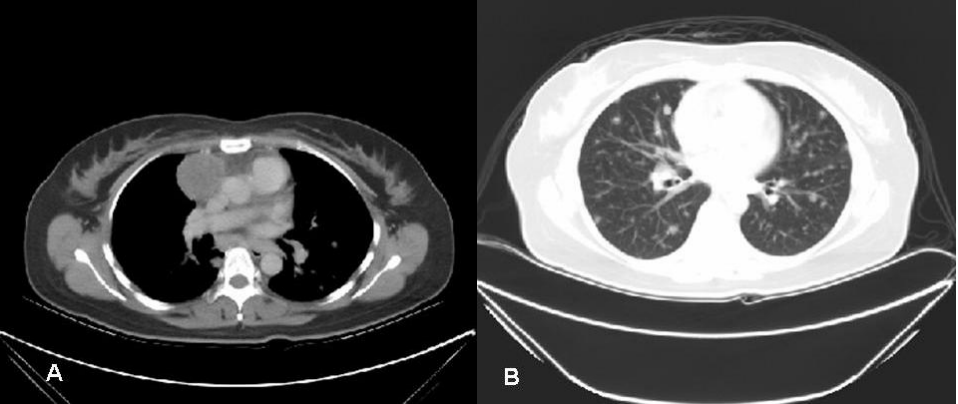
Chest CT scan. A. a right anterior mediastinal shadow. B. multiple pulmonary nodular lesions.

A 42 year-old woman presented with intermittent dry cough of ten months duration. No fever, chest tightness, bone pain, anorexia, dyspnea on exertion or body weight loss was noted. She took some medicines at a local clinic to control her cough. However, blood-tinged sputum appeared one month prior to hospital admission. No bone pain or lymphadenopathy was noted on examination. Her breathing was slightly coarse without rales or rhonchi on auscultation. A chest X-ray (Figure 1) and computed tomography (CT) (Figure 2A, B) revealed a right anterior mediastinal shadow with multiple pulmonary nodular lesions.

Tracing back her history, she was a non-smoker, without occupational or environmental exposure to air pollutants or micro-organisms. No family members had any similar clinical manifestations nor had any died of cancer before. No related travel history was noted during this time.

After admission, a bronchoscopic examination revealed no endobronchial lesion or abnormal secretions. A pulmonary function test showed a mild restrictive ventilatory defect. The whole body bone scan was negative for tumor involvement. A CT-guided biopsy was recommended but the patient refused to undergo this procedure. The hemogram, leukocyte differentiation count, and coagulating profile were all within normal ranges.

**Figure 3:**
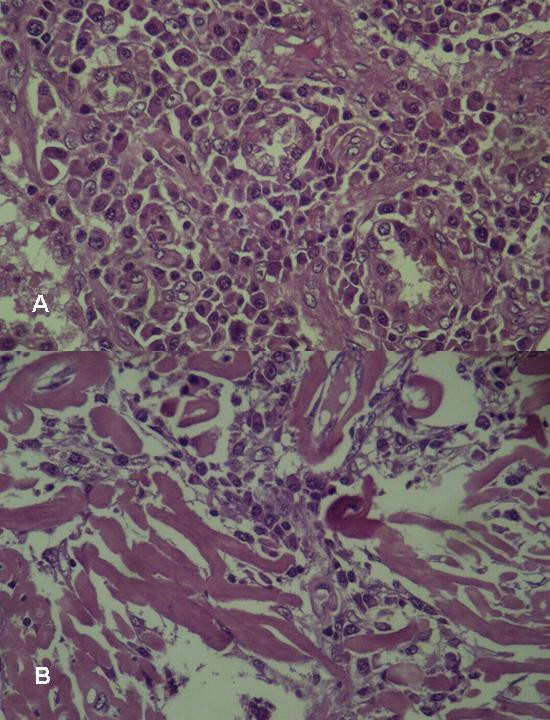
Microscopically, abnormal plasma cells infiltrate in the lung parenchyma (A) and abnormal plasma cells accompanied with amyloid (B) (H & E, 400×).

**Figure 4:**
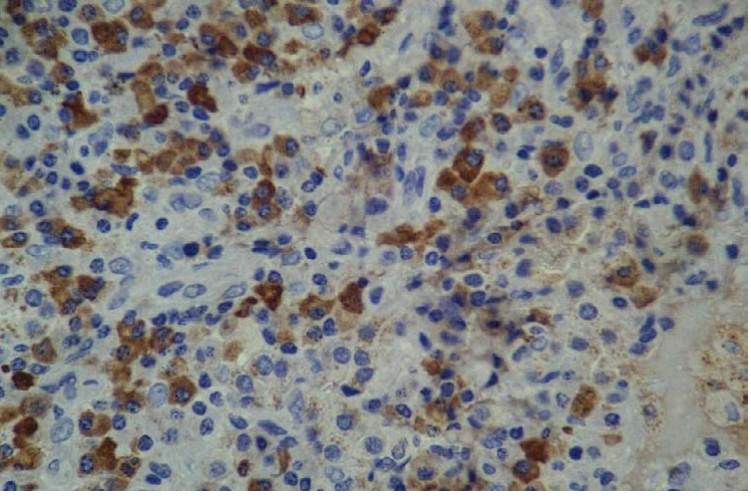
These tumor cells stained positively for kappa light chains (400×).

A biopsy through video-assisted thoracoscopic surgery (VATS) was then indicated to confirm the diagnosis. A 3 cm working incision and a 1 cm scope port were designed for performing this procedure. Removing a wedge of lung tissues including the masses from two separate sites were performed smoothly and the microscopic examination showed a solid mass made up mostly of plasma cells (Figure 3). These tumor cells stained positively for kappa light chains (Figure 4), but negatively for lambda chains.

After confirming the diagnosis, the patient received a series of related evaluations such as serum calcium, urine Bence-Jones protein and plasma electrophoresis for M protein detection. However, all of the above examinations were negative. The skull, spine and pelvis X-ray revealed no osteolytic lesions. The bone marrow biopsy revealed normal patterns of cell distribution.

She received adjuvant chemotherapy, as originally planned, including mephalan and steroids, and her symptoms improved after two months of treatment.

## Discussion

Plasma cell neoplasm can be classified into the following types: multiple myeloma (bone marrow and other systemic involvements), solitary myeloma (bone plasmacytoma), extramedullary (soft tissue) plasmacytoma, and plasmablastic sarcoma [4]. Extramedullary plasmacytoma (EMP), which belongs to the category of non-Hodgkin's lymphoma, is present in about 3% of all plasma cell neoplasms [4-7]. It is defined as a soft-tissue plasma cell tumor occurring in the absence of systemic signs of multiple myeloma, such as bone osteolytic lesions, plasma cell infiltration in bone marrow, lytic bone lesion, or serum or urine myeloma protein (M-component) [1]. EMP affects males three to four times more often than females, with an average age of 55. However, one third of patients with EMP are under 50 years old [1,6,8]. In a comprehensive literature search reviewing over 700 patients with EMPs, the EMPs were located predominantly (over 80%) at the upper aeordigestive tract. Pulmonary or pleural EMPs, which was first reported by Gordon and Walker [9], only occurred in twelve of them [2]. Their presentations varied, from a single pulmonary nodule (most commonly in the hilum, the upper lobe of the lung, and both sides of the lungs equally affected), a lobar or segmental consolidation, to bilateral diffuse infiltrates [2,10-14]. However, none of them had clinical pictures or image findings similar to our presented case (a mediastinal mass with bilateral multiple pulmonary nodules).

Diagnosis of undetermined pulmonary nodular lesions can be accomplished by transbronchial, CT- or sono- guide needle biopsy, as well as surgical biopsy through the VATS or open thoracotomy [15,16]. VATS has been increasingly applied for the diagnosis or treatment of undetermined solitary or multiple pulmonary nodule(s) because it is more accurate in diagnosis than needle biopsy and less invasive in treatment than the open procedure [17].

The EMP should be differentiated from other plasma cell tumors. As described in the above paragraph, there should be no systemic signs to exclude the possibility of multiple myeloma. In addition, the EMP should also be differentiated from other types of plasma cell tumor, such as reactive plasmacytoma and plasma cell granuloma, or lymphoma (MALT, marginal and immunoblastic) [7,18]. Although the absence of M-spike on plasma electrophresis cannot differentiate EMP from benign granuloma, [14], the clinical characteristics such as the size of the main mass and the numerous pulmonary seeding nodular lesions favor the diagnosis of malignant EMP. The best method to differentiate EMP from other types of plasma cell tumors or lymphomas is that the EMP is positive for CD38 and monoclonal cytoplasmic light chain expression of malignant plasma cells obtained by surgical or needle biopsy.

Like other plasma cell tumors, EMPs are highly radiosensitive with 80 to 100% of patients successfully achieving local control and with a 50 to 65% ten-year disease-free survival rate [19,20]. Surgery can be considered for localized EMPs located in areas other than the head and neck, such as the gastrointestinal tract [2]. Localized EMPs can also be effectively controlled by irradiation [21]. Multiple disseminated pulmonary plasmacytoma, such as in our presented patient, can be effectively controlled by different regimens of combined chemotherapeutic agents, such as mephalan and steroids, VMCP or VAMP, as described in other literature [22-24]. The prognosis of patients with EMP is usually better than those with multiple myeloma. The EMPs, unlike solitary myeloma, advances to multiple myeloma only in a minority of cases [12]. The local recurrence rate is about 10–30%, with a 17–48% progression to multiple myeloma and the median survival of about 63–101 months [24].

## Conclusion

We presented a case of EMP with a mediastinal mass with multiple pulmonary nodules. After reviewing the literature, this has never before been reported as a clinical manifestation of EMP.

## Competing interests

The author(s) declare that they have no competing interests.

## Authors' contributions

**SPL **– literature review and write clinical part of this manuscript.

**YSL **– pathological diagnosis, collaborate the completion of the pathological part of this manuscript.

**CHT **– pathological diagnosis and write pathological part of this manuscript.

**TCT **– completion of the clinical part of the manuscript

All authors read and approved the manuscript
